# ASO Author Reflections: Cholangitis Following Pancreatoduodenectomy

**DOI:** 10.1245/s10434-025-18463-7

**Published:** 2025-10-08

**Authors:** Abedin Suljagic, Oskar Swartling, Ebba Seiler Henningson, Christoph Ansorge, Stefan Gilg, Ernesto Sparrelid, Poya Ghorbani

**Affiliations:** 1 Division of Surgery and Oncology, Department of Clinical Sciences, Intervention and Technology, Karolinska Institutet, Stockholm, Sweden; 2 Department of Surgery, Nyköping County Hospital, Nyköping, Sweden; 3 Department of Medicine, Clinical Epidemiology Division, Karolinska Institutet, Stockholm, Sweden; 4 Department of Surgical Sciences, Uppsala University, Uppsala, Sweden; 5 Department of Upper Abdominal Diseases, Karolinska University Hospital, Stockholm, Sweden

## Past

Postoperative cholangitis is a recognized complication following pancreatoduodenectomy and is most often related to biliary strictures or reflux at the hepaticojejunostomy. It is associated with increased morbidity and mortality in patients operated on for malignant or benign disease.^1,2^ Identifying risk factors and outcomes is essential to support the development of standardized prevention and treatment protocols, which are currently lacking. Previous studies have highlighted potential risk factors. For example, a recent report demonstrated that a bile duct diameter <5 mm significantly increased the risk of postoperative cholangitis.^3,4^ However, further data on prevalence, risk factors, and long-term outcomes remain limited.

## Present

This study investigated postoperative cholangitis in patients undergoing pancreatoduodenectomy in Stockholm, Sweden.^5^ The long-term follow-up was facilitated by the universal healthcare system and the use of unique personal identity numbers. Cholangitis episodes were identified using the established Tokyo criteria. Interestingly, patients with preoperative jaundice or those who required biliary drainage before surgery were less likely to develop postoperative cholangitis, possibly because of the preoperative bile duct enlargement. In contrast, patients who developed other postoperative complications, such as bile leakage, had a significantly higher risk of subsequent cholangitis. Notably, preoperative cholangitis was not associated with an increased risk of postoperative episodes. The study also demonstrated that cholangitis may recur even several years after surgery, emphasizing the importance of long-term surveillance.

## Future

Given the relatively high frequency of postoperative cholangitis, further research is warranted to identify strategies that reduce its occurrence. Future efforts should focus on refining pre- and intraoperative techniques to minimize the risk of hepaticojejunostomy strictures and reflux. Optimizing postoperative management is equally important, both for the treatment of acute episodes and for the prevention of recurrence. The establishment of standardized treatment protocols—including recommendations on timing of intervention, antibiotic regimens, and structured follow-up—would represent an important step toward improved patient outcomes.
